# Group antenatal care (gANC) for Somali-speaking women in Sweden – a process evaluation

**DOI:** 10.1186/s12884-022-05044-9

**Published:** 2022-09-21

**Authors:** Malin Ahrne, Ulrika Byrskog, Birgitta Essén, Ewa Andersson, Rhonda Small, Erica Schytt

**Affiliations:** 1 Department of Women’s and Children’s Health, Karolinska Institutet, Stockholm, Sweden; 2 School of Health and Welfare, Dalarna University, Falun, Sweden; 3 Women’s and Children’s Health, IMCH, Uppsala University, Uppsala, Sweden; 4 Judith Lumley Centre, La Trobe University, Melbourne, Australia; 5 Centre for Clinical Research Dalarna, Uppsala University, Falun, Sweden; 6 Faculty of Health and Social Sciences, Western Norway University of Applied Sciences, Bergen, Norway

**Keywords:** Group antenatal care, Maternal and child health, Antenatal care, Pregnancy, Migration, Somali-born women, Complex interventions, Process evaluation, Inequity

## Abstract

**Background:**

Language supported group antenatal care (gANC) for Somali-born women was implemented in a Swedish public ANC clinic. The women were offered seven 60-min sessions, facilitated by midwives and starting with a presentation of a selected topic, with an additional 15-min individual appointment before or after. The aim of this study was to assess the feasibility for participants and midwives of implementing The Hooyo (“mother” in Somali) gANC intervention, including implementation, mechanisms of impact and contextual factors.

**Methods:**

A process evaluation was performed, using The Medical Research Council (MRC) guidelines for evaluating complex interventions as a framework. A range of qualitative and quantitative data sources were used including observations (*n* = 9), complementary, in-depth and key-informant interviews (women *n* = 6, midwives *n* = 4, interpreters and research assistants *n* = 3) and questionnaire data (women *n* = 44; midwives *n* = 8).

**Results:**

Language-supported gANC offered more comprehensive ANC that seemed to correspond to existing needs of the participants and could address knowledge gaps related to pregnancy, birth and the Swedish health care system. The majority of women thought listening to other pregnant women was valuable (91%), felt comfortable in the group (98%) and supported by the other women (79%), and they said that gANC suited them (79%). The intervention seemed to enhance knowledge and cultural understanding among midwives, thus contributing to more women-centred care. The intervention was not successful at involving partners in ANC.

**Conclusions:**

The Hooyo gANC intervention was acceptable to the Somali women and to midwives, but did not lead to greater participation by fathers-to-be. The main mechanisms of impact were more comprehensive ANC and enhanced mutual cultural understanding. The position of women was strengthened in the groups, and the way in which the midwives expanded their understanding of the participants and their narratives was promising. To be feasible at a large scale, gANC might require further adaptations and the “othering” of women in risk groups should be avoided.

**Trial registration:**

The study was registered in ClinicalTrials.gov (Identifier: NCT03879200).

## Background

Carefully evaluated antenatal care (ANC) interventions are needed to address maternal and reproductive health inequalities for migrant women [1–4]. Somali-born migrant women have demonstrated higher rates of severe pregnancy complications [5–9]. Qualitative studies [3, 10–12] and systematic reviews [4, 13, 14] show that migrant women more often experience communication problems [4, 14–16], lack of familiarity with care systems [4, 14, 15], sub-optimal care [4, 13, 15, 17, 18] and discrimination [4, 14, 16, 17]. In Sweden, migrant women [19] including Somali-born women [6], commence ANC later, make fewer ANC visits and are less likely to contact their obstetric care provider for decreased fetal movements [5] than their Swedish-born counterparts. Lower attendance in childbirth preparation and parental classes during pregnancy among migrant women in Sweden and elsewhere has also been reported [20–22].

Few attempts have been made to develop and evaluate interventions for improving health outcomes for Somali-speaking and other migrant women during pregnancy and birth in Sweden, for example using mobile applications to improve communication and bi-lingual doulas [23, 24]. All in all, Somali-born women constitute a sub-group of women in Sweden with elevated risk for poor reproductive health outcomes, and because of the relatively large number of Somali migrants in Sweden, many clinics have sufficient numbers of Somali women attending to consider strategies to improve their care.

### Standard ANC in Sweden

Standard ANC in Sweden for uncomplicated pregnancies includes 8–9 individual midwife appointments, free of charge [25], with referral to a physician if needed. ANC uptake is close to 100% and very few women have fewer than four ANC visits. Language interpreting can be arranged face-to-face or by telephone. Optional birth preparation and parent education is provided individually and in groups/seminars. A first, early ANC visit focusing on lifestyle factors in gestational week (gwk) 6–8 has been gradually introduced since 2017. The second visit (gwk 10–12) is usually 45 min, including a detailed patient history, with the midwife who becomes the “designated midwife” throughout pregnancy, to secure continuity of care and patient safety. Remaining appointments are usually 30 min. Ultrasound for pregnancy dating is recommended in gwk 19–20 [25]. Visits 3–9 include pregnancy check-ups and provision of information about e.g. pregnancy, labour, birth and parenting. Two postnatal check-ups are recommended, at 10 days and 6–12 weeks after birth. Normal tasks of midwifery clinics include ANC, follow-up visits after labour, contraceptive counselling and screening for cervical cancer. Although not clearly articulated in guidelines and policies [25], the Swedish norm is inclusion of partners in birth preparation and ANC [26].

### Group antenatal care

Group antenatal care (gANC) is an ANC-model which typically integrates pregnancy check-ups with group sessions for education and peer support in groups of pregnant women [27, 28]. Slightly different gANC models have been developed in different settings [29–31]. Particularly for women at risk of adverse outcomes, gANC has demonstrated potential to increase attendance, improve satisfaction with care and pregnancy outcomes such as lower rates of preterm birth, increased breastfeeding rates and reduce risk for depressive symptoms [32–35]. Swedish gANC interventions have not previously focused on migrant women [36]. Inspired by other models, the Hooyo (“mother” in Somali) gANC intervention was developed, implemented and evaluated with a participatory approach from 2016–2020. Midwives and representatives from the Somali-Swedish community were part of a project reference group [37], and initial focus group discussions (FGDs) explored experiences of standard care and provided input on the intervention design, as described below [10, 37].

### The Hooyo gANC intervention

From the third ANC appointment around gwk 20, women were offered seven 60-min language-supported group sessions together with 6–8 other women + fathers-to-be or other partner, facilitated by midwives, starting with a presentation of a selected topic. Frequency and total number of appointments followed the Swedish national ANC recommendations, as did the topics that were covered (life style, pregnancy, birth, practical birth preparations, the newborn baby, infant feeding, parenthood and relationships) [25]. Pregnancy controls were performed by each woman’s designated midwife during a 15-min individual appointment scheduled adjacent to the group session.

The Hooyo gANC intervention has been evaluated in a quasi-experimental study, using historical controls to assess impact, with the two primary outcomes being *overall ratings of care* and *emotional wellbeing* measured by the Edinburgh Postnatal Depression Scale (EPDS) (Submitted). In summary, no differences in the overall ratings of antenatal care were identified, but women in gANC were happier with the information they received on different aspects of pregnancy, labour and birth.

### Initial assumptions and underlying principles

The study protocol describes initial assumptions and underpinning principles, including a logic model with hypothesised mechanisms of effect and desired outcomes [37]. Language-supported gANC was expected to provide peer support, more comprehensive antenatal care with integrated childbirth preparation and parent education, foster a greater understanding of the healthcare system and improve communication and mutual understanding, thus empowering women and reducing bias among care providers. Person Centred Care (PCC) and women-centred maternity care [38, 39] were underpinning principles, based on previous research on what migrant women expect from ANC [14, 39, 40] and on initial focus group discussions (FGDs) [10]. Motivational Interviewing (MI) adapted for groups was recommended as a tool to support this [41]. A bilingual female assistant nurse who was also a trained interpreter facilitated the sessions, instead of only having an accredited interpreter present.

Inclusion of partners was a desired outcome, based on findings from the FGDs with Somali parents [10] and on previous Swedish gANC interventions [42]. We also hypothesized that gANC could potentially be cost-effective and enable more time with the midwife for the participants.

Two clinics were involved in the intervention development. The design and hypothesised mechanisms of impact differed slightly between the two. At one site we hypothesised that language-supported gANC would improve Swedish-Somali women´s experiences of ANC and emotional wellbeing, in groups with only Somali-born women. This intervention arm was implemented. At the other site, we hypothesised that gANC with women of diverse ethnic backgrounds including Swedish (“mixed groups”) would have additional positive impact on integration and increased mutual understanding across language and cultural barriers. This latter site withdrew prior to implementation however, for a range of reasons (see Results).

This was the first time gANC was implemented in Sweden with the specific purpose of trying to improve ANC for a group of women with migrant background, so it was important to explore acceptability and feasibility of the intervention, both among pregnant women and care providers. The aim of this process evaluation was to explore aspects of providing and receiving language-supported gANC, from the perspective of both Somali-born women and midwives.

## Methodology

### Study design

The Medical Research Council (MRC) framework for performing process evaluations of complex interventions developed by Moore et al. [43] was used. The following key functions were investigated:

- The implementation process, including how successful the delivery of the intervention was and what was actually delivered including fidelity to what had been planned, the adaptations that were made and who were reached and not reached by the intervention.
- Mechanisms of impact, including participant responses to and interactions with the intervention, mediators and unexpected pathways and consequences. [43]

### Setting

The implementing clinic was public, with 10 midwives and one manager, situated in a mid-sized Swedish city (approx. 50,000 inhabitants). The catchment area is diverse including a large Somali community. Childbirth and parent education is integrated in the individual visits, and in addition, a 3-h evening seminar for prospective parents is offered, however, in Swedish only. In 2018, ANC enrolment in the catchment in average area was done in gwk 11 [44]. The Swedish target is 80 patients/midwife, and the national average for public clinics was 85 in 2019 [45].

### Description of the data

Data collection for this process evaluation was nested in the different project phases of the main study. Informants were Somali-born women who attended gANC, the midwives and interpreters implementing the intervention, and research assistants who facilitated implementation. Qualitative and quantitative data were collected from multiple sources employing different measurement tools and activities: observations of sessions, complementary, in-depth and key-informant interviews, field notes, open-ended questions in questionnaires to participating women and midwives and logbooks, questionnaires and midwives’ brief evaluation form documenting each session. The MRC framework for performing process evaluations of complex interventions guided the development of interview guides and other data collection tools. Table 1 describes the range of data collection methods used.

**Table 1:** Overview of data sources and description of data

Data source	Description of data
*Qualitative data*	
Observations	Semi-structured observations of gANC sessions (in total nine by authors MA (*n* = 7), UB (*n* = 1), MA & RS (*n* = 1))
Interviews	•Complementary interviews with participants in gANC (*n* = 6) •In-depth interviews with midwives (*n* = 4) •Key-informant interviews with interpreter/research assistants (*n* = 3)
Field notes	•Notes from interviews and meetings with the research group, reference group, clinic staff etc •Additional field notes e.g. from outreach activities
Questionnaires	•Open-ended questions in questionnaires (women *n* = 44; midwives *n* = 8)
*Quantitative data*	
Logbook	Attendance in group sessions
Questionnaires	•Participants in gANC; 2 months after birth (*n* = 44) •Midwives; post-intervention questionnaire (*n* = 8)
Evaluation form for sessions	Completed by the midwives after each session (*n* = 50)

### Recruitment and analyses of different data sources

#### Interviews

A purposeful recruitment strategy was used for all interviews, and interview guides were developed for the different types of interviews. We applied a pragmatic approach to saturation of data, because of the large total amount of data sources. The research assistant contacted gANC participants for complementary interviews, based on her pre-understanding of the participants’ views of gANC that she gained from the main study questionnaires, as we wanted to interview participants with both positive and less positive experiences. The interview guide contained questions such as whether it was good with a Somali group or if a mixed group would have been better, if the partner had attended and reasons for not attending, if the participant made new friends through the group and if she would like to attend gANC again. The participant interviews aimed to provide more in depth understanding of a small, but diverse number of women’s views of Hooyo than could be gathered in the main study questionnaires. The complementary interviews were performed by MA together with a research assistant, in the home of the participant (*n* = 2) and by telephone (*n* = 4).

The recruitment of midwives for in-depth interviews was also purposeful, aiming at eliciting a broad spectrum of experiences, and was done by the research team. The interviews were recorded, transcribed and analysed using content analyses with an inductive approach and the reporting of the findings is aligned with the MRC framework for undertaking complex interventions [46, 47]. The interview guide contained questions on the dialogue with and between parents-to-be, patient safety, attitude towards Somali-born parents, fathers’/partners’ role etc. The 45–60 min interviews were performed face-to-face by MA and BE.

#### Observations

Ethnographic methodology was used in the observations, to provide holistic and rich insights into the behaviours, actions and viewpoints of the groups [48]. Ethnographic methods have been usefully employed in other studies exploring midwife-patient interaction [49]. An observation protocol was developed, and extensive notes were taken. We made observations of sessions with various midwives and both early and later in each cycle of sessions.

#### Quantitative data from questionnaires, protocols and the logbook

Recruitment of women for the main study was undertaken by midwives and the bilingual research assistants and is presented in detail elsewhere [37]. This process evaluation includes quantitative data collected as part of the controlled historical evaluation of gANC (submitted). Data from questionnaires, evaluation forms and the logbook are presented with descriptive statistics.

#### Ethical considerations

This study was carried out in accordance with the Helsinki Declaration. Ethical approval was obtained from the Stockholm Ethical Review Board (2015/1703–31/1) (main study) and from the Swedish Ethical Review Authority [2019–01116] (process evaluation). Oral and written information about the study, its voluntary nature and confidentiality was provided in Swedish and Somali, and written consent was provided by all informants.

## Results

**Fig. 1:**
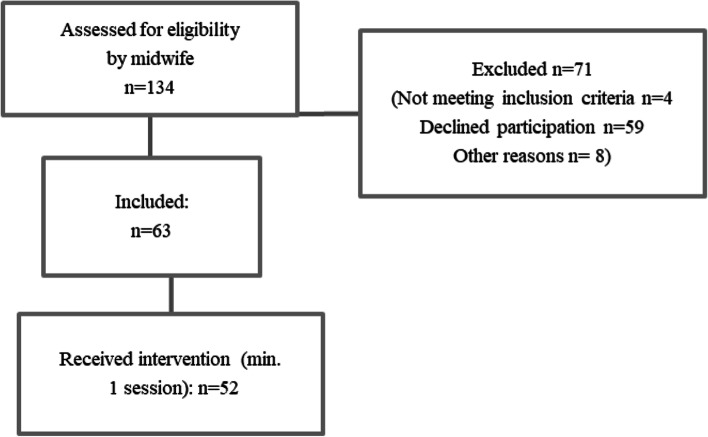
Flowchart of the number of women recruited to gANC

Findings from both qualitative and quantitative data sources are presented under headings that mirror our initial assumptions, underlying principles and the MRC framework for performing process evaluations of complex interventions. In this study, seven series of gANC sessions (*n* = 50 sessions) were arranged in a meeting room in the ANC clinic during an 18-month period. In total, 63 women were recruited to gANC and 52 took part in at least one session, which is described in Fig. 1. Of all eligible women who were invited, excluding those who did not meet the inclusion criteria, 45% declined participation in the intervention and received standard care.

On average, each session was attended by 4.2 women (range 1–8). The mean number of sessions attended per woman was 3.8. When women were absent, another individual appointment was scheduled. Only four women attended all sessions.

The midwives (*n* = 9) were all female, of Swedish/Nordic ethnicity, above 46 years of age, and all with more than 5 years of experience as midwives.

### Contextual factors that influenced implementation of gANC

The midwives, including management at the implementing site, were instrumental in initiating the project and supportive of the study. The second clinic was also enthusiastic about their involvement. After running a first pilot of group sessions, the second clinic withdrew due to a key staff member’s extended sick-leave, heavy workload and the introduction of a regional plan to streamline and standardize ANC, which proved not compatible with the implementation of gANC.

gANC was thus implemented in one clinic for 18 months, during which further contextual changes took place. Updated regional clinical guidelines on female genital mutilation/cutting (FGM/C) were introduced in October 2017 [50]. The clinic also recruited a bilingual assistant nurse (Somali-Swedish) who supported day-to-day activities such as routine pregnancy controls. She became an important resource for improving the dialogue between midwives and Somali-speaking patients.

### Implementation

#### Was gANC implemented according to plan?

During the design phase, the research team and the reference group discussed a number of design options. Table 2 summarises key considerations made prior to the intervention, the intervention as it was intended and finally as it was actually implemented (fidelity). The main deviations related to fidelity were that partners did not attend to the extent that we had intended and many sessions were facilitated by a single midwife. Only two groups had a follow-up session after birth with their babies, but those sessions were much appreciated by the women.

**Table 2:** Considerations, intervention as intended and intervention as delivered

Key considerations prior to implementation	Intervention as intended	Intervention as delivered (fidelity)
Optimal group size (women)	6–8 women	4.2 women/session (range 1–8)
Number and length of sessions	Seven 60-min sessions.	Six-seven 60-min sessions.
Follow-up session after birth with babies?	Not “compulsory”, but suggested and discussed during implementation as optional.	Two groups (of seven) had a follow-up session after birth with their babies.
Time of day for sessions	Weekdays AM	Sessions were scheduled on weekdays (AM), which was considered convenient by participants and midwives, but not optimal for those with daytime jobs or studying.
Partners	To be invited to attend all sessions. However, women had the opportunity to decide otherwise.	Four men attended at least one session. Most groups (women and midwives together) decided not to invite men.
Content of sessions	Selected topics to be presented (suggested in manual). After that, open for questions and discussion. Participants encouraged to raise any concerns and identify topics of interest.	Each session started with a presentation of a selected topic. Aids commonly used were pictures, anatomical models and displaying objects. Films on pregnancy and childbirth, some available online in Somali, shown in 1/3 of sessions Topics “pregnancy”, “birth” and “the newborn baby” were frequently discussed. “Parenthood” and “relationships” less frequent (in 4 of 50 sessions).
Other professionals invited	Child health nurses, physiotherapists etc. optional.	None invited.
Should clinical assessments be integrated in the sessions or not?	No	Clinical assessments were conducted in private adjacent to the sessions by women’s designated midwife, who was not necessarily one of the midwives leading the group.
Interpreting	Interpreter/"cultural broker” assigned to all groups.	A bilingual female interpreter who also facilitated sessions was present in all but a few sessions.
Sessions facilitated by midwives	Preferably two midwives—was believed optimal to facilitate dialogue and good group dynamics.	Half of sessions facilitated by one midwife only. Reasons provided were “few women attending” and a heavy workload. Some midwives (*n* = 3) preferred to facilitate sessions alone.
Open or closed session groups (i.e. possibility to shift between groups and have fluid starting and end-dates)?	Closed groups, i.e. the same individuals in every session.	Only closed groups – women were assigned to a particular group to give them a better chance of getting to know each other and facilitated the administration for midwives.
Tools to support person-centering in groups	Use of MI techniques in the groups (such as midwives asking open-ended questions and being “reflective in their listening and response”).	MI techniques were used to some extent.

In the observed sessions, the topics appeared to be seen as relevant by women. During the planning phase, midwives had mentioned that non-pregnancy related problems, like concerns for other family members or housing, were frequently brought up in ANC. This was not observed in the group session.

Midwives sometimes considered “violence”, “relationships” and “FGM/C” difficult to discuss in groups, but what was considered sensitive was not always what the midwives had expected.

”Some women are very private, there are things you don´t discuss in a group. But to talk about circumcision and examinations during labour in the group, that was not a problem at all.” (Midwife interview 2).

Despite an initial ambition to use a more informal pedagogy with a main emphasis on dialogue, both the observations and interviews revealed a tendency towards a more classroom-like situation:

”We had one group where everybody got seated in a row, it really became like a school-bench, and then I tried to re-arrange, like, let’s move around so that we face each other, but it takes some time to get a hold of those things.” (Midwife interview 4).

#### Who were reached and not reached by the intervention?

Both primiparous (*n* = 11) and multiparous women (*n* = 52) were recruited, and at baseline, 24% of the women were grand multiparas (≥ 5 births). Women’s mean age was 31 years and the median length of stay in Sweden was seven years. Half (50.8%) the women had < 6 years of education, 22.2% had 7–9 years and 22.2% had > 10 years of education. The majority, 78.8%, were living with a partner.

Our findings suggest that gANC reached women with relatively low Swedish-proficiency. Swedish-proficiency (self-reported, including speaking, writing and understanding) was “well or fluent” in 65% of the women. When the midwives first informed women and asked about their interest in gANC, their impression was that women with fluency in Swedish were not interested. In the second recruitment step, when the research assistant provided more in-depth information, a frequently mentioned motive for declining participation was that gANC was too time-consuming. Other reasons suggested in follow-up interviews with some participants were that women may not have understood what gANC was, felt they were already sufficiently informed about pregnancy, labour and birth or preferred not to discuss private matters in groups, or a combination of these. No baseline data were collected for the women who declined participation.

#### Inclusion of partners

The groups set their own rules, and some groups decided not to invite partners. In total, only four men attended any session. It is notable that partners seemed more likely to attend the individual appointments before or after the group session. Sometimes spouses waited in the waiting room during group sessions. In the post-intervention questionnaire, 58% (*n* = 25) of the female participants believed that it would have been valuable for fathers to participate in the group sessions. The research assistants were also approached by women who highlighted the importance of targeting men more directly. However, 42% (*n* = 18) said that male presence would have made them feel embarrassed. Other reasons for low male participation suggested by women were more practical, for example that evening sessions would be more feasible for some partners. Low male attendance was noticed and discussed during the course of implementation. The midwives reported that they did not push the agenda that partners should attend. This was also confirmed in the observations, where the absence of partners was not highlighted or addressed. Some midwives expressed the opinion that exclusive women’s groups could provide “a sheltered zone”, where women could be empowered through peer-support.

“Actually, I was ambivalent to… I mean, I think there should be some sessions where men are actively invited, but then there is a need for this”sisterhood”, where women feel that now we are a group of women that sit and chat, and then we can talk freely about anything. I think both are needed.” (Midwife interview 4).

#### Patient safety and individual appointments

Prior to the intervention the reduced time with the “designated midwife” was a concern among the midwives, for fear that patient safety could be affected due to a loss of overall patient responsibility and control. Post-intervention, the midwives believed the possibility to identify pregnancy related risk factors remained the same in gANC (*n* = 6), had improved (*n* = 1) or was more difficult (*n* = 1) compared to standard care. However, half of the midwives (4/8) thought “caring for the emotional wellbeing of women” became more difficult in gANC.

The individual 15-min appointment adjacent to the group session was often extended to 30 min, because the midwives felt that 15 min was too short. Additional appointments were sometimes scheduled to compensate for the reduced individual time. Among participants, 13 (30%) said they would have liked more private time with the midwife.

Negative features of gANC mentioned in midwife interviews were that “private time” with the midwife was important for this target group, and that it was difficult to capture individual issues in gANC. An illustrative example from a group observation was when a midwife told an anxious woman with a lot of questions: “*Don´t worry, I will give you a private lesson.”* (Observation 5).

#### Was the intervention acceptable to women and midwives?

The majority of women in the post-intervention questionnaire thought listening to other pregnant women during the sessions was valuable (*n* = 41; 91%) and 42 women (98%) felt comfortable in the group. Most participants (*n* = 34; 79%) said they felt supported by the other women, and that gANC suited them (*n* = 34; 79%). One woman said that the midwife was just providing information which she could easily find herself elsewhere (interview 5), and some believed gANC took too much time (*n* = 7; 16%).

There were a range of views about attending gANC again for a future pregnancy. One primiparous woman wanted to attend gANC if she got pregnant again because *“it was so much fun”*, and she would have more experiences to share (interview 2). Another first-time mother on the other hand had appreciated gANC, but would not like to attend again:

“No. I know a lot now, I don´t need to attend again” (Woman, interview 1).

From the women´s perspectives, positive features of gANC together with other Somali-born women included the possibility of getting pregnancy and childbirth information in Somali, and the opportunity to share experiences with other women who share a common language, especially if Swedish proficiency was inadequate.

The midwives reported being “happy with the session” in 41/50 sessions. Positive features of gANC identified by the midwives was the opportunity for women to receive more information, to meet other parents-to-be and to share knowledge and advice with each other – and with the midwife. Yet only two midwives thought gANC increased work efficiency (2/8), four answered “no” (4/8) and two “to some extent” (2/8). Midwives believed gANC was time-consuming and they got frustrated when participants were absent.

#### Alternative models

In the complementary interviews with women, a preference for, or interest in, gANC with mixed ethnic groups was expressed, even though it was believed to be a situation of “win some, lose some”. Possible language barriers could be overcome. In mixed groups it might be harder to make new friends, but the perceived advantages of mixing might outweigh the downsides. Perceived benefits with mixed groups were to get the perspectives of others, to know how others think about pregnancy and childbirth. Pregnancy could provide an opportunity to meet women outside the Somali community—pregnancy providing a common shared experience. One participant said that she did not have so much in common with the others in the Somali group, who she felt shared previous experiences that she could not relate to:

“It was OK [with the Somali group]. It…would have been better if it was a mix, which would have been better. I wanted to hear more from people who had given birth here, people with more experience from here, as I don´t have, I mean, I don´t have any such memory from the home country and I don´t have any experience from there, so it felt like I was, I don´t know, a bit excluded you can say” (Woman interview 5).

Five midwives wanted to continue with gANC – two with the current model and three in a modified form with fewer sessions – and three did not want to continue. When discussing alternative ANC models, some of the midwives expressed ambivalent feelings about language supported “Somali-groups” vs. mixed ethnicity groups. The midwives thought mixed groups could support integration and that the information provided to parents-to-be would become more equal. There was a concern that when tailoring information too much, there is a risk of losing valuable pieces of information, which can affect the quality. One midwife thought that the greater value placed on mixed groups was more in the eyes of society rather than of the participants.

“Yes, I have thought of that, and I think maybe it’s I who would want it to work, because that´s how we want our society [to be]…” (Midwife interview 3).

### Mechanisms of impact

#### Social interactions in the groups

The observations revealed a generally relaxed, respectful and accepting atmosphere during the sessions. Groups differed, some were quieter while others were livelier. Dialogue and interaction were generally encouraged by the midwives. For example, participants vividly shared recipes and advice on nutritious food to support lactation and recovery after birth in one session. Differences in how midwives welcomed and introduced participants and themselves, offered refreshments and organized seating were noticed; seemingly simple things that could still influence comfort levels and in turn group dynamics. In one session, some participants sat with their jackets on and appeared uncertain, but subsequently they relaxed and joined in the dialogue (observation 1). Humor was used as a tool to create a relaxed, informal atmosphere and enhanced dialogue.

“..it was amazing how they listened and were attentive, and we could laugh about things that had been misunderstood, I mean, everybody was just very open, midwives and the pregnant women too” (Midwife interview 3).

A participant touched upon the issue of how the group format was supportive, and it also happened that participants asked questions on behalf of someone else in the group.

”When you are in a group, you get more time, and if I forget to ask something, somebody else might remember to ask” (Woman; free text in questionnaire)

#### Communication and interpreting

Communication between midwives and participants, and with the interpreter, was assessed as mostly smooth in observations. The majority of women (*n* = 39; 91%) believed they got enough space in the groups to talk about what was important to them.

From the midwives’ perspective, some, but not all, thought communication with participants improved, one strong reason being that the sense of mutual understanding increased:

”Communication got much better because the women opened up more and revealed more in the group. One could understand more about their situation both in their home country and here in Sweden.” (Midwife, open-ended questionnaire question).

The midwives who were less positive about communication in gANC were concerned about discussing private matters in groups, and lack of control over what kind of information is delivered when more than one midwife is involved.

Moreover, interpreting in gANC differed from that in standard care. The interpreter in gANC also helped to facilitate the sessions, for example by explaining things and making an effort to include quieter women. Most participants were happy with the interpreting and the role of the interpreter. A few (*n* = 8; 19%) thought the interpreting was somewhat disruptive.

#### gANC more comprehensive

Midwives mostly felt that the gANC participants were happy to have gained more knowledge and information, and that the group format had improved women’s agency. One knowledge gap that was revealed was pelvic floor exercises, recognized by both participants and midwives. Other examples of areas where participants had gained new knowledge were anatomy and birth control.

“It struck me that even though these women have lived here quite a long time, some have had several children in Sweden, knowledge gaps were revealed when they got more “in control”. We have not reached out enough with information.” (Midwife interview 2).

The same midwife described a shift in power balance, similar to her experiences from engaging in outreach community work:

”There is a different power balance when you are invited, when I am invited, and it is not in my own comfort zone, but I reach out to the women instead. The groups have been like that.” (Midwife interview 2).

One midwife noted that to gain and sustain trust, becoming better at explaining was a key, and gANC could provide a good platform for this.

”We screen all children for jaundice, yes, we do it all the time, but it seems we have not been able to tell the women what we are doing. They come, show their children, probably wondering what we are doing, but it is not clear to them what the purpose of the control is. Perhaps it is the same with Swedish speakers too, that they just submit to the routines, I don´t know. I see gANC as a way to get a good reputation, perhaps I can put it that way. Should I listen to my neighbor or to the health care provider? Then, if some have a positive experience of gANC, that can spread…” (Midwife interview 2).

#### Person-centering in the group

The midwives were ambivalent about how person-centering was achieved in gANC. It became both easier and more difficult to provide person-centred care. The system with “designated midwives” was challenged with gANC, and the midwives struggled to merge the two models. One midwife said that it was *“difficult to know if the patient has received adequate information”* with gANC. Another commented:

“It was easier with those in the group who were my own patients, then one could continue individually, if they had a need to ask more, afterwards…” (Midwife, open-ended questionnaire)

On the other hand, women could wish to confide in a midwife who was not her designated midwife, and gANC could trigger issues that participants wanted to discuss privately later, and that might not have been revealed in standard ANC.

Experience and confidence might be required to enable midwives to let go of some of these feelings of loss of control:

”I am not so worried to let go of some control, because I am thinking that I have worked for a long time, so I know what things I need to include to feel they get the information they are supposed to get. It is ok that the group is buzzing…- That is when the interesting things happen.” (Midwife, interview 2).

Sometimes it was clear that the midwives had not gained a sufficient understanding of the participants’ previous experiences and knowledge level in the sessions. For example, after having attended several sessions a woman asked *“Are you midwives?”* (observation 3). She thought midwives worked at the delivery ward only, and the midwives had not introduced themselves adequately enough. Sometimes midwives overrated levels of health literacy and struggled to explain what were to them “self-evident” ideas, like the importance of having “good shoes” to prevent back pain.

#### Increased knowledge and understanding – for midwives too

Both midwives and participants learned and developed new insights and skills through gANC. In one session, the midwife listed contraceptives on the white board. After quite some time, a participant asked *“Which of these can be inserted?”* The midwife realized that a central piece of the information had been missed and she adapted accordingly by fetching demonstrating materials (observation 6). Another example of new insights was the perceptions related to epidural analgesics:

”Epidural for example. Some thought the side effects could be hair growth and back injuries. I thought it was more that they wanted to have a natural birth. I knew that there was also a fear about an injection in the back, but not that there were so many non-medical ideas about what could happen.” (Midwife interview 3).

The experiences from gANC may also have influenced how the midwives provided individual care:

“Yes, I have changed the way I provide information in private sessions after the gANC. One has picked up a lot, like, hey, this I have to discuss more…” (Midwife interview 3)

One initial assumption was that gANC would be less prejudice prone and reduce bias, because of the format and the changing power dynamics. Expressions of stereotypes were rarely observed in gANC.

Prior to the intervention, the midwives expressed some concerns about certain cultural and religious norms, for example related to pregnant women fasting during Ramadan. In some sessions, a reluctance, hesitancy or uncertainty about discussing cultural norms relevant for maternal health was observed – “missed opportunities”. To exemplify, in one session, (observation 4) preparations for Eid al-fitr were discussed by participants. The midwives did not take the opportunity to follow up on this and ask questions about fasting or diet during pregnancy.

#### The research assistants as mediators in implementing gANC

Two research assistants were employed during different periods of the study, and a third research assistant worked short-term in the project. The recruitment and retaining of participants was challenging and time-consuming. The research assistants played an important role in community outreach work promoting the intervention, in disseminating information about the project in the local Somali community and served as a liaison between midwives, women attending gANC and the research team. The research assistants engaged with women at a community-based play group. They had regular contact with participants, sometimes phoning them before group sessions to confirm their attendance or reminding them to come, and they frequently served as informal interpreters for conversations between midwives and patients.

## Discussion

Language-supported gANC was implemented in one clinic with overall fidelity to the design. The intervention was acceptable and feasible to participants, provided that it was voluntary and in combination with adequate individual time with the midwives. The majority of attending women said that gANC suited them, they felt comfortable and supported by the other women in the group, however, attendance was fairly low. gANC was also largely acceptable to midwives who noted that enhanced knowledge and cultural understanding had contributed to more women-centred care, but gANC might require further adaptations to be feasible on a larger scale and to be more time efficient. Many of the eligible women declined participation, which might indicate that gANC does not suit everyone, or also that new models of care take time to promote and become accepted. In addition, few women attended all sessions, and the reasons for being absent were not systematically collected, partly to respect the integrity of the participants, which was a limitation. Further, the intervention was unsuccessful in involving partners in ANC.

A strength of this process evaluation was the participatory approach taken, from development of gANC to its implementation and evaluation, with the Somali-Swedish research assistants and the interpreter playing important roles. This evaluation reflects the perspectives of pregnant women, midwives and interpreters and is intended to give a holistic view of the possible mechanisms of effect of gANC.

A more comprehensive ANC, with information and education alongside pregnancy controls, provided a good opportunity for women and midwives to get to know each other and strengthened the position of women through the group format. Language-supported gANC filled an existing knowledge gap and contributed to more equitable ANC for the participants, particularly as the childbirth and parent education provided as part of standard care was not an option for those with limited Swedish-proficiency. How social support in gANC can facilitate learning and more positive relationships with health care providers has been described previously in qualitative studies of gANC [51, 52]. The midwives were comfortable in the educator role, and the participants were probably more familiar with a student role, and expected that as well, so the pedagogy of the sessions became more”traditional” than was initially envisaged, but it is likely that with time, the midwives would have become increasingly confident in guiding gANC sessions.

Despite our intentions to include fathers, few partners attended. Our qualitative study prior to the intervention showed that Somali-Swedish men were facing barriers to inclusion in ANC such as unclear expectations from the health care providers, and that they wished to be more involved [10]. These challenges for fathers-to-be seem to have remained with gANC. Female participants in the present study noted that male inclusion would have been valuable, but when the decision not to invite partners was agreed they possibly adjusted to the minority who had expressed feeling uncomfortable about male attendance. We did not explore reasons for not wanting men to participate. Midwives were reluctant to overrule participants who were in favour of not inviting men to the sessions. In other studies, a more neo-assimilatory discourse has been described, where promoting “Swedish values” including gender equity has been seen as important by health professionals [53, 54]. However, a racialised discourse, perceiving and portraying spouses as dominating and/or over-protective has also been described, which may partly explain the reluctance of midwives to actively invite men [53]. The conclusion from a Swedish study of father´s satisfaction with gANC compared to standard care was that both models could be improved to attract men [55]. Our study suggests that improving different ANC care models might be even more important for the inclusion of fathers-to-be with a migrant background. At the same time, achieving both stronger female peer support and stronger male inclusion in the same gANC model might be difficult and more research in this area is suggested.

From the midwives’ perspective, perceived added values with gANC were more comprehensive ANC, providing a platform that could generate trust, which is central for compliance, person-centering and patient safety [39]. This is in line with findings from other studies on gANC showing increased midwife satisfaction with providing care [29]. However, in Swedish standard ANC women are usually assigned a “designated midwife” with personal responsibility for individual women, something associated with patient safety. The midwives were reluctant to interfere with that system and to reduce the individual time spent with women in order to safeguard patient safety, and they adapted strategies such as booking additional shorter individual appointments. Similar experiences of challenges with merging different models have been described in a study of gANC and caseload midwifery [56], where it was perceived as if the two models collided. This possible barrier to introducing gANC on a larger scale in Sweden might require more consideration.

Person-centred care [39] was an underpinning principle of this intervention, and may not be immediately seen as consistent with gANC but our findings suggest that it is possible and that it was achieved, for example through increased mutual understanding and that women and midwives got better acquainted. However, it may be challenging and require time and practice for service providers. The personal narrative constitutes the starting point for person-centering and should be the foundation for the relationship between midwife and pregnant woman [38]. Understanding each woman’s personal narrative and prior experiences more thoroughly was identified as a need in our gANC intervention. Even though this was achieved with the combination of gANC and individual care, it may not have been the case in the group sessions alone.

The midwives expanded their understanding of the participants through the dialogue encouraged in gANC, giving them new insights and a more nuanced picture of Somali-Swedish women, which in turn may help to reduce possible bias. In the US context it has been suggested that group antenatal care may impact on racial health inequities primarily through its impact on *clinicians* and on the systems in which they work [57]. Consequently, our findings support the idea that gANC had an added value for the clinicians, as well as for the participants.

We initially hypothesized that language-supported groups would strengthen and empower participants, and that “mixed groups” would strengthen integration. Unfortunately, it was not possible to compare the planned different intervention arms, as one study site withdrew. However, some of the women who attended the language-supported gANC said that they would have preferred mixed groups. Even though some benefits might be lost (like shared language), they suggested that there might be even more to gain from mixed groups. At the same time, single language groups seemed to strengthen and empower the participants. Ethnicity may become a demarcation line that differentiates people, and interventions targeting ethnic groups may inadvertently contribute to notions of otherness, despite good intentions, and regardless of whether ethnic groups are viewed by staff as more “exciting”, or alternatively as more “problematic” [53]. Therefore, the relevance for a particular sub-group should be carefully considered before initiating language-supported gANC, and person-centering should be safe-guarded.

Our intervention is in line with the framework for quality maternal and newborn care (QMNC) which highlights the need for preventive and supportive care that works to strengthen women’s capabilities in the context of respectful relationships, is tailored to their needs, focuses on promotion of normal reproductive processes, and in which first-line management of complications and accessible emergency treatment are provided when needed [58]. However, the QMNC Framework also suggests that viewing pregnancy and childbirth through a ‘risk lens’ should be avoided, and proposes a distinction between ‘what all women and babies need’ and ‘what some women and babies also need’, which is in line with the concept of proportionate universalism, describing how health inequalities can be addressed within universal systems [59]. While tailoring care to individual needs, the group format can still provide added value in antenatal education and care, if it is combined with a person-centred approach. Our findings suggest that language-supported gANC suited most women and may be helpful in addressing inadequate health literacy and in empowering women. At the same time, we recognize that forming groups based on language or ethnicity may also be problematic and, if not carefully considered, may contribute to the othering of migrant women.

It would have been valuable to include the perspectives of fathers-to-be but that was not possible. Another limitation was a lack of data for exploring why women chose standard care rather than participating in gANC, and to find out more about why many women only attended a few sessions. Even though the intervention was only implemented in one site, the findings can still inform others who want to address health inequalities during pregnancy for migrant women by developing gANC or designing new models. Future gANC intervention studies should investigate the optimal number of group sessions. Perhaps fewer sessions would be of similar value, and not be considered as time-consuming by both participants and midwives.

## Conclusions

The Hooyo gANC intervention was largely acceptable to participants and midwives but did not engage fathers-to-be. The main mechanisms of impact were more comprehensive ANC and enhanced mutual cultural understanding. The position of women was strengthened in the groups, and the way in which the midwives expanded their understanding of the participants and their narratives was promising. To be feasible on a large scale, language-supported gANC might require further adaptations and the “othering” of women in risk groups should be avoided.

## Data Availability

The datasets used and/or analysed during the current study are available from the corresponding author on reasonable request.

## Abbreviations

ANC: Antenatal care
EPDS: Edinburgh Postnatal Depression Scale
FGD: Focus group discussions
gwk: Gestational week
gANC: Group antenatal care
MRC: Medical Research Council
MI: Motivational Interviewing
PCC: Person Centred Care
QMNC: Quality Maternal and Newborn Care
SC: Standard care
SGA: Small for gestational age
WHO: World Health Organization
